# Histone H3.5 forms an unstable nucleosome and accumulates around transcription start sites in human testis

**DOI:** 10.1186/s13072-016-0051-y

**Published:** 2016-01-15

**Authors:** Takashi Urahama, Akihito Harada, Kazumitsu Maehara, Naoki Horikoshi, Koichi Sato, Yuko Sato, Koji Shiraishi, Norihiro Sugino, Akihisa Osakabe, Hiroaki Tachiwana, Wataru Kagawa, Hiroshi Kimura, Yasuyuki Ohkawa, Hitoshi Kurumizaka

**Affiliations:** Laboratory of Structural Biology, Graduate School of Advanced Science and Engineering, Institute for Medical-oriented Structural Biology, Waseda University, 2-2 Wakamatsu-cho, Shinjuku-ku, Tokyo, 162-8480 Japan; Division of Transcriptomics, Medical Institute of Bioregulation, Kyushu University, 3-1-1 Maidashi, Higashi-ku, Fukuoka, Fukuoka 812-8582 Japan; Department of Biological Sciences, Graduate School of Bioscience and Biotechnology, Tokyo Institute of Technology, Yokohama, 226-8501 Japan; Faculty of Medicine and Health Sciences, Yamaguchi University, 1-1-1 Minami-Kogushi, Ube, 755-8505 Japan; Program in Chemistry and Life Science, School of Science and Engineering, Meisei University, 2-1-1 Hodokubo, Hino, Tokyo 191-8506 Japan

**Keywords:** Histone variant, Nucleosome, Chromatin, Testis, Spermatogenesis, Transcription start site

## Abstract

**Background:**

Human histone H3.5 is a non-allelic H3 variant evolutionally derived from H3.3. The H3.5 mRNA is highly expressed in human testis. However, the function of H3.5 has remained poorly understood.

**Results:**

We found that the H3.5 nucleosome is less stable than the H3.3 nucleosome. The crystal structure of the H3.5 nucleosome showed that the H3.5-specific Leu103 residue, which corresponds to the H3.3 Phe104 residue, reduces the hydrophobic interaction with histone H4. Mutational analyses revealed that the H3.5-specific Leu103 residue is responsible for the instability of the H3.5 nucleosome, both in vitro and in living cells. The H3.5 protein was present in human seminiferous tubules, but little to none was found in mature sperm. A chromatin immunoprecipitation coupled with sequencing analysis revealed that H3.5 accumulated around transcription start sites (TSSs) in testicular cells.

**Conclusions:**

We performed comprehensive studies of H3.5, and found the instability of the H3.5 nucleosome and the accumulation of H3.5 protein around TSSs in human testis. The unstable H3.5 nucleosome may function in the chromatin dynamics around the TSSs, during spermatogenesis.

## Background

In eukaryotes, genomic DNA is organized into chromatin, which is tightly packaged but allows replication, recombination, repair, and transcription [1]. The nucleosome, which accommodates about 150 base pairs of DNA, is the fundamental structural unit of chromatin [2]. The four core histones, H2A, H2B, H3, and H4, are the protein components of the nucleosome. These core histones contain a conserved histone-fold domain [3]. Specific dimer formation occurs between H2A and H2B (H2A-H2B), and between H3 and H4 (H3-H4) [2–4]. During nucleosome formation, two H3-H4 dimers are first assembled on DNA, where they form a subnucleosomal structure called the tetrasome [1–5]. Two H2A-H2B dimers are then incorporated into the tetrasome, to form the mature nucleosome [1].

For histones H2A, H2B, and H3, non-allelic isoforms have been identified as histone variants [6]. Eight histone H3 variants, H3.1, H3.2, H3.3, H3T (H3.4), H3.5, H3.X, H3.Y, and CENP-A (CenH3), exist in humans [7–18]. These histone H3 variants have distinct expression profiles during the cell cycle and in different tissues, suggesting their specific functions. H3.1 and H3.2 are produced in S-phase cells, and are incorporated into chromatin during DNA replication [5, 14, 15, 19]. In contrast, H3.3 is expressed throughout the cell cycle, and can be used as a replacement for histone H3 during transcription and DNA repair [5, 14, 15, 20, 21]. In addition, H3.3 may be involved in defining specific chromatin domains, such as heterochromatin, euchromatin, telomeres, and centromeres [21–24]. H3.X and H3.Y are expressed in normal and malignant tissues, including brain, and H3.Y is induced by stress stimuli, such as nutrient starvation [17]. CENP-A is an essential component of the chromosomal centromere [7], and forms the fundamental centromeric nucleosome [25]. H3T is highly expressed in the testis, suggesting that the H3T nucleosome may function during spermatogenesis [6, 8, 9]. H3T forms nucleosomes in vitro, and was incorporated into chromatin when ectopically expressed in human cells [26]. However, the H3T nucleosome was extremely unstable in vitro, and H3T tagged with green fluorescent protein (GFP) was rapidly exchanged in living cells [26].

H3.5 is conserved among great apes and Neanderthals, but not in non-hominid primates [18]. The H3.5 mRNA is highly expressed in the human testis [18]. In cells, ectopically expressed H3.5 is reportedly incorporated into chromatin, and predominantly localized in the euchromatic region [18]. Ectopic H3.5 expression complemented the growth defect of H3.3 knockdown cells, suggesting that it has an overlapping function with H3.3, as a replacement histone [18]. However, endogenous H3.5 has not been detected at the protein level, and the biochemical and cellular functions of the H3.5 nucleosome have not been clarified so far.

In the present study, we performed comprehensive studies of human histone H3.5, including structural and biochemical analyses with reconstituted nucleosomes, fluorescence recovery after photobleaching analyses with living cells, immunohistochemical analyses with human testis sections, and chromatin immunoprecipitation followed by sequencing (ChIP-Seq).

## Results

### The H3.5 nucleosome is unstable

**Fig. 1:**
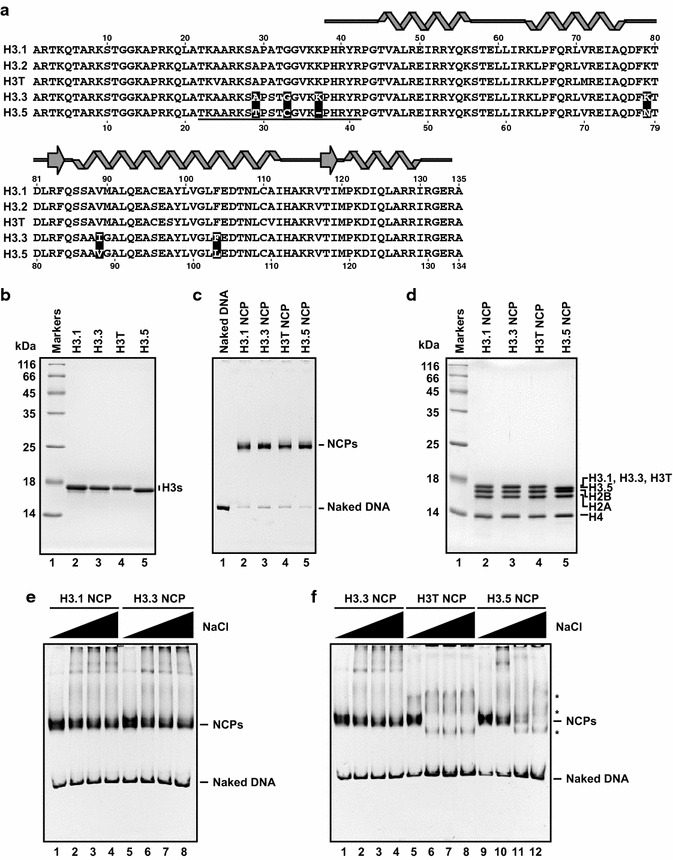
The nucleosome containing histone H3.5 is unstable. **a** Sequence comparison between human H3.1, H3.2, H3.3, H3T, and H3.5. The amino acids in H3.5 that differ from those in H3.3 are indicated by *black boxes* with white characters. The epitope peptide sequence used to generate the H3.5 antibody is underlined. The α-helices and β-strands found in the crystal structures of the human nucleosomes are represented on the top of the panel. **b** 18 % SDS-PAGE analysis of purified histones H3.1, H3.3, H3T, and H3.5, stained with Coomassie Brilliant Blue (CBB). **c** Non-denaturing 6 % PAGE analysis of purified nucleosomes containing H3.1, H3.3, H3T, and H3.5, stained with ethidium bromide. *Lane 1* represents the naked DNA used in the nucleosome reconstitution. Nucleosome core particles are denoted by NCPs. **d** Histone compositions of the purified nucleosomes containing H3.1, H3.3, H3T, and H3.5, analyzed by 18 % SDS-PAGE with Coomassie Brilliant Blue staining. **e** Salt resistance assays of the H3.1 and H3.3 nucleosomes and **f** the H3.3, H3T, and H3.5 nucleosomes. Bands corresponding to nucleosomes are indicated by NCPs. *Asterisks* represent bands corresponding to non-nucleosomal DNA-histone complexes [26]

Human histone H3.5 lacks the lysine residue present at position 37 in the major H3 variants, H3.1, H3.2, and H3.3 (Fig. 1a). An amino acid sequence alignment suggested that H3.5 is more similar to H3.3, than to H3.1 and H3.2. As compared to H3.3, H3.5 has five amino acid differences at positions 29, 33, 78, 88, and 103. The Thr29, Cys33, Asn78, Val88, and Leu103 residues of H3.5 correspond to the Ala29, Gly33, Lys79, Ile 89, and Phe104 residues of H3.3, respectively (Fig. 1a). To reveal the biochemical properties of H3.5, we purified H3.5 and other H3 variants, including H3.1, H3.3, and H3T (Fig. 1b), and then reconstituted nucleosomes containing each of the human H3 variants with H2A, H2B, and H4, by the salt dialysis method (Fig. 1c). Like the other H3 variants, H3.5 formed nucleosomes with the core histone ratio of 1:1:1:1 (Fig. 1c, d).

We next tested the stability of the H3.5 nucleosome, using a salt-titration assay. The reconstituted nucleosomes were incubated at 50 °C for 1 h, in the presence of 0.4, 0.6, 0.7, or 0.8 M NaCl, and the resulting nucleosomes were analyzed by native polyacrylamide gel electrophoresis. In this assay, the H3.1 and H3.3 nucleosomes were equally stable, and formed nucleosomes in 0.4–0.8 M NaCl (Fig. 1e). In contrast, the intact H3.5 nucleosome was only detected under the 0.4 M and 0.6 M NaCl conditions (Fig. 1f, lanes 9 and 10). At higher NaCl concentrations (i.e., 0.7 and 0.8 M), the bands corresponding to the H3.5 nucleosome disappeared, indicating that the H3.5 nucleosome was disrupted (Fig. 1f, lanes 11 and 12). Consistent with the previous study [26], the H3T nucleosome was disrupted in 0.6 M NaCl, and was the most labile (Fig. 1f, lanes 5–8). We previously purified the complexes corresponding to the bands remaining after the H3T nucleosome disruption, and confirmed that these bands were non-specific H2A-H2B-DNA complexes (Fig. 1f, asterisks) [26]. These results showed that the H3.5 nucleosome is more stable than the H3T nucleosome, but is clearly unstable as compared to the H3.1 and H3.3 nucleosomes. The formation of unstable nucleosomes may be a common feature of the human testis-specific H3 variants.

### Crystal structure of the H3.5 nucleosome

**Fig. 2:**
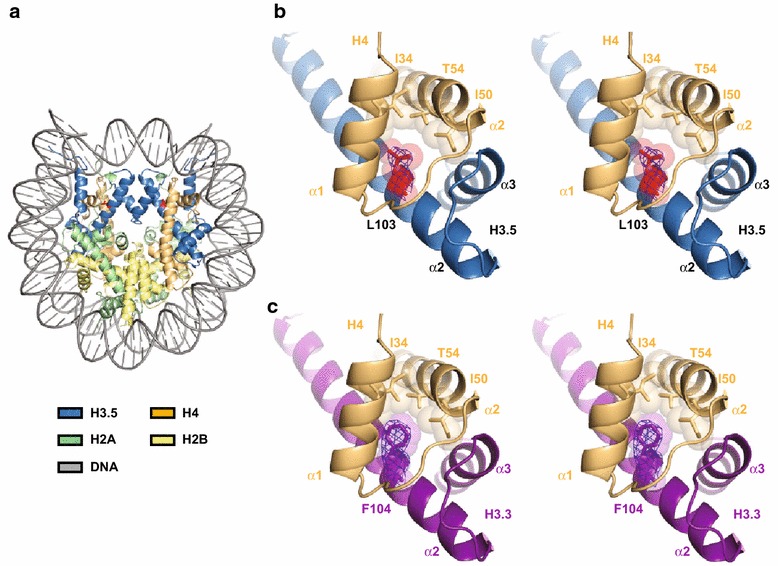
Crystal structure of the H3.5 nucleosome. **a** Overall structure of the H3.5 nucleosome. The H3.5, H4, H2A, H2B, and DNA molecules are colored *sky blue*, *light orange*, *pale green*, *pale yellow*, and *gray*, respectively. The H3.5-specific Leu103 residues are colored *red*, and their side chains are represented. **b** Stereo view of the H3.5 (*sky blue*) and H4 (*light orange*) region around the H3.5 Leu103 residue (*red*). The 2mFo-DFc electron density map around the H3.5 Leu103 residue is shown as a blue mesh, contoured at 1.5σ. The van der Waals surfaces of the H3.5 Leu103 side chain atoms, and the H4 Ile34, Ile50, and Thr54 side chain atoms, are represented. **c** Stereo view of the H3.3 (*deep purple*) and H4 (*light orange*) region around the H3.3 Phe104 residue in the H3.3 nucleosome structure [PDB:3AV2] [27]. The 2mFo-DFc electron density map around the H3.3 Phe104 residue is shown as a *blue* mesh, contoured at 1.5σ. The van der Waals surfaces of the H3.3 Phe104 side chain atoms, and the H4 Ile34, Ile50, and Thr54 side chain atoms, are represented

To understand the structural basis for the instability of the H3.5 nucleosome, we determined the crystal structure at 2.8 Å resolution (Fig. 2a; Table 1). The overall structure was similar to that of the H3.3 nucleosome [27], as expected. H3.5 contains two residues, Asn78 and Leu103, which are not conserved in H3.3. Both residues do not directly interact with either the H2A-H2B dimers or the DNA, which could possibly affect nucleosome stability. Leu103, however, is located at the interface of H3.5 and H4, and may possibly exhibit reduced hydrophobic interactions compared with that of H3.3 (Fig. 2b, c). In H3.3, the corresponding residue is Phe104, which fills the pocket created by the α1 and α2 helices of H4, and apparently forms hydrophobic interactions with the side chains of the H4 Ile34, Ile50, and Thr54 residues [27]. In contrast, such close hydrophobic interactions are not observed around the Leu103 residue in the H3.5 nucleosome, because Leu has a smaller side chain than Phe (Fig. 2b). These data suggested that this structural difference may account for the instability of the H3.5 nucleosome.

**Table 1:** Summary of data collection and refinement statistics

	H3.5 nucleosome
Data collection	
Space group	*P*2_1_2_1_2_1_
Cell dimensions	
a, b, c (Å)	104.89, 109.13, 174.49
α, β, γ (°)	90.00, 90.00, 90.00
Resolution (Å)	50.00–2.80 (2.90–2.80)
*R*_sym_	9.4 (50.0)
*I/σI*	10.2 (2.7)
Completeness (%)	99.5 (96.5)
Redundancy	8.8 (6.4)
Refinement	
Resolution (Å)	37.81–2.80
No. reflections	49,924
*R*_work_/*R*_free_	22.8/26.9
No. atoms	
Protein	5919
DNA	5980
*B*-factors	
Protein	66.4
DNA	132.5
R.m.s. deviations	
Bond lengths (Å)	0.005
Bond angles (°)	0.815

One crystal was used for data collection. Values in parentheses are for highest-resolution shell

### The Leu103 residue is responsible for the instability of the H3.5 nucleosome

To test whether the H3.5 Leu103 residue contributes to the instability of the H3.5 nucleosome, we performed a mutation analysis. There are five amino acid differences between H3.3 (Ala29, Gly33, Lys79, Ile89, and Phe104) and H3.5 (Thr29, Cys33, Asn78, Val88, and Leu103) (Fig. 1a). Since the residue corresponding to Lys37 (or Lys36) of H3.3 is missing in H3.5, the H3.5 Asn78, Val88, and Leu103 residues correspond to the H3.3 Lys79, Ile89, and Phe104 residues, respectively (Fig. 1a). We did not target the Thr29, Cys33, and Val88 residues of H3.5 for mutagenesis, because they were unlikely to contribute to the instability of the H3.5 nucleosome, as the H3.5 Thr29 and Cys33 residues are located in the flexible N-terminal tail region, and the H3.5 Val88 residue is conserved in H3.1 (Fig. 1a).

**Fig. 3:**
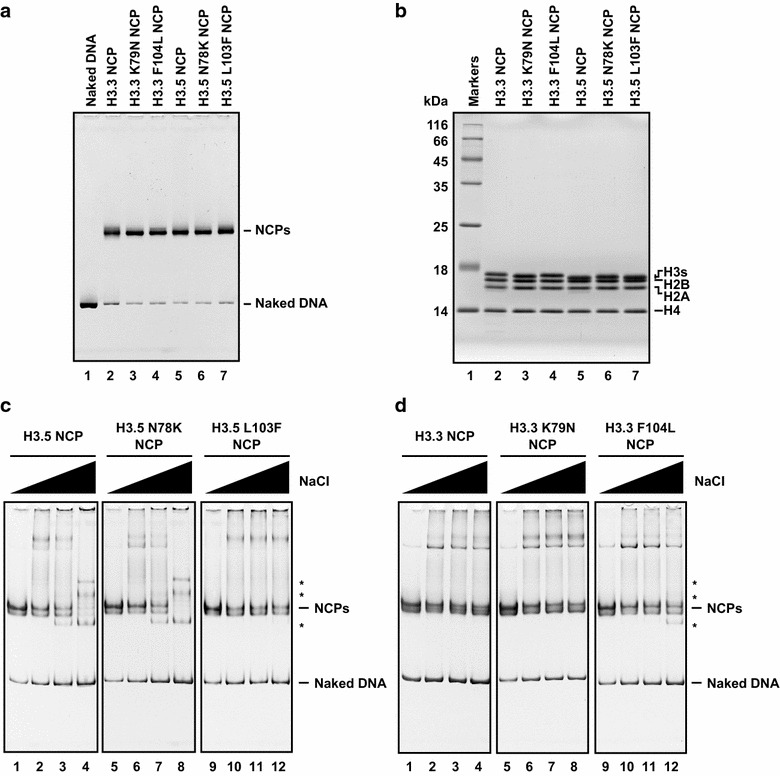
Mutational analysis of H3.5 and H3.3. **a** Non-denaturing 6 % PAGE analysis of the purified nucleosomes containing H3.5 and H3.3 mutants, stained with ethidium bromide.* Lane 1* represents the naked DNA used in the nucleosome reconstitution. Nucleosome core particles are denoted by NCPs. **b** Histone compositions of the purified nucleosomes containing H3.3 and H3.5 mutants, analyzed by 18 % SDS-PAGE with Coomassie Brilliant Blue staining. **c** Salt resistance assays of the H3.5 mutant nucleosomes, and **d** the H3.3 mutant nucleosomes. Bands corresponding to nucleosomes are indicated as NCPs. *Asterisks* represent bands corresponding to non-nucleosomal DNA-histone complexes [26]

We therefore prepared two H3.5 mutants, N78K and L103F, in which the H3.5 Asn78 and Leu103 residues were replaced with the corresponding H3.3 residues, Lys and Phe, respectively. We also prepared two H3.3 mutants, K79N and F104L, in which the H3.3 Lys and Phe were reciprocally substituted with the corresponding H3.5 residues. These amino acid substitutions in H3.3 and H3.5 did not affect nucleosome formation by salt dialysis (Fig. 3a, b).

**Fig. 4:**
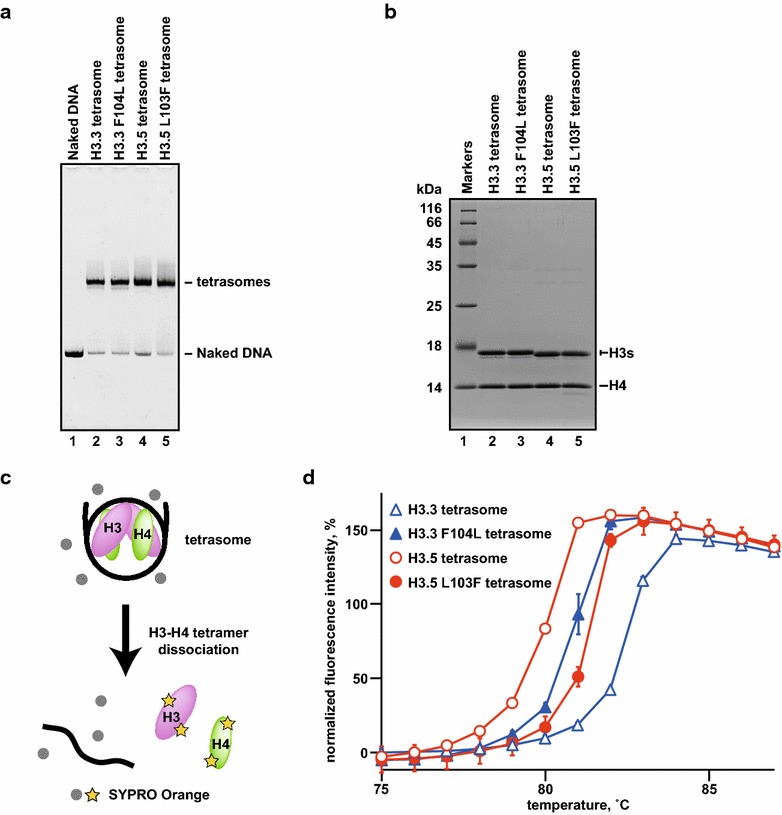
Thermal stability of tetrasomes. **a** Purified tetrasomes containing H3.3 (*lane 2*), H3.3 F104L (*lane 3*), H3.5 (*lane 4*), and H3.5 L103F (*lane 5*) were analyzed by non-denaturing 6 % PAGE analysis. The gel was stained with ethidium bromide. *Lane 1* represents the naked DNA used for the reconstitution of tetraosomes. **b** Histone compositions of the purified tetrasomes. Purified tetrasomes containing H3.3 (*lane 2*), H3.3 F104L (*lane 3*), H3.5 (*lane 4*), and H3.5 L103F (*lane 5*) were analyzed by 18 % SDS-PAGE with Coomassie Brilliant Blue staining. **c** The schematic representation of thermal stability assay. **d** The thermal stability curves for the tetrasomes containing H3.3 (*open triangles*), H3.3 F104L (*closed triangles*), H3.5 (*open circles*), and H3.5 L103F (*closed circles*) are presented. Three experiments were independently performed and averages of these results were plotted with standard deviation values

We next performed a salt-titration assay with the nucleosomes containing the mutant H3.5 and H3.3 under the same conditions as in Fig. 1f, in the presence of 0.4, 0.6, 0.7, or 0.8 M NaCl. The H3.5 N78K nucleosome and the wild-type H3.5 nucleosome were similarly unstable (Fig. 3c, lanes 1–8). In contrast, the H3.5 L103F nucleosome remained intact at higher NaCl concentrations (Fig. 3c, lanes 9–12). Complementarily, the H3.3 F104L nucleosome became less stable (Fig. 3d, lanes 9–12), as compared to the wild-type H3.3 and H3.3 K79N nucleosomes (Fig. 3d, lanes 1–8). To confirm that the H3.5-specific Leu103 residue directly weakens the DNA binding of the H3-H4 complex, we reconstituted tetrasomes, in which the H3.5-H4, H3.3-H4, H3.5 L103F-H4, or H3.3 F104L-H4 tetramer complex wraps the DNA (Fig. 4a, b). We then performed the thermal stability assay [28]. In this assay, the thermal dissociation of histones from the DNA can be monitored as a fluorescence signal (Fig. 4c). As shown in Fig. 4d, the H3.5 tetrasome was disrupted at lower temperatures than the H3.3 tetrasome, indicating that H3.5 associates with DNA more weakly than H3.3. This is consistent with the H3.5 instability observed in the salt-titration assay (Figs. 1, 3). Interestingly, the H3.5 tetrasome instability was partially compensated by the H3.5 L103F mutation (Fig. 4d). Consistently, the H3.3 F104L mutation reduced the tetrasome stability (Fig. 4d). These data support the above notion that the H3.5-specific Leu103 residue, which structurally reduces the hydrophobic interaction with H4, is at least partially responsible for the instability of the H3.5 nucleosome.

### Higher mobility of GFP-tagged H3.5 in living cells

**Fig. 5:**
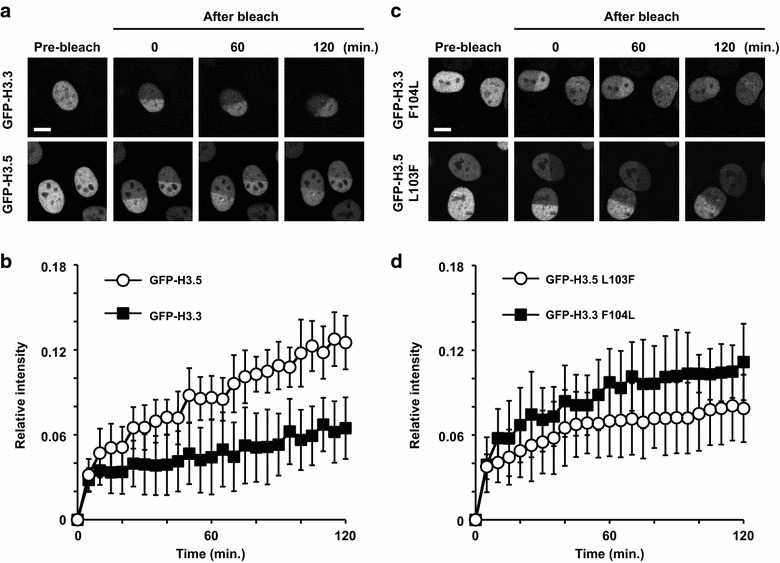
Mobility of GFP-H3.5 and GFP-H3.3 in living cells. **a** FRAP analysis of HeLa cells expressing GFP-H3.3 or GFP-H3.5. *Bar* indicates 10 μm. **b** The mobility of GFP-H3.3 or GFP-H3.5 in living cells, analyzed by bleaching one-half of the nucleus. The averages of the relative fluorescence intensity of the bleached area were plotted with the standard deviations (*n* = 10–11). **c** FRAP analysis of HeLa cells expressing GFP-H3.3 F104L or GFP-H3.5 L103F.* Bar* indicates 10 μm. **d** The mobility of the GFP-H3.3 F104L or GFP-H3.5 L103F mutant in living cells, analyzed by bleaching one-half of the nucleus. The averages of the relative fluorescence intensity of the bleached area were plotted with the standard deviations (*n* = 10–11)

To examine whether H3.5 is incorporated into nucleosomes less stably than H3.3 in living cells, we performed fluorescence recovery after photobleaching (FRAP), using HeLa cells expressing GFP-H3.5 and GFP-H3.3 [26, 29, 30]. One-half of the nucleus was bleached, and the fluorescence intensity was measured in the presence of cycloheximide, to suppress the fluorescence recovery due to new protein synthesis. As shown in Fig. 5a, both GFP-H3.5 and GFP-H3.3 exhibited slow recovery, consistent with their incorporation into chromatin as H3.1-GFP [31]. Quantitative measurements then indicated that GFP-H3.5 recovered substantially faster than GFP-H3.3 (Fig. 5b), suggesting that nucleosomal H3.5 exchanges more rapidly than H3.3.

FRAP analyses of the mutants further revealed that the GFP-H3.5 L103F mutant recovered more slowly than the reciprocal GFP-H3.3 F104L mutant (Fig. 5c, d). These results indicated that the H3.5-specific Leu103 residue is critical for the rapid exchange of H3.5 in living cells, in good agreement with the in vitro salt-titration data.

### Presence of human histone H3.5 in testicular cells within seminiferous tubules

**Fig. 6:**
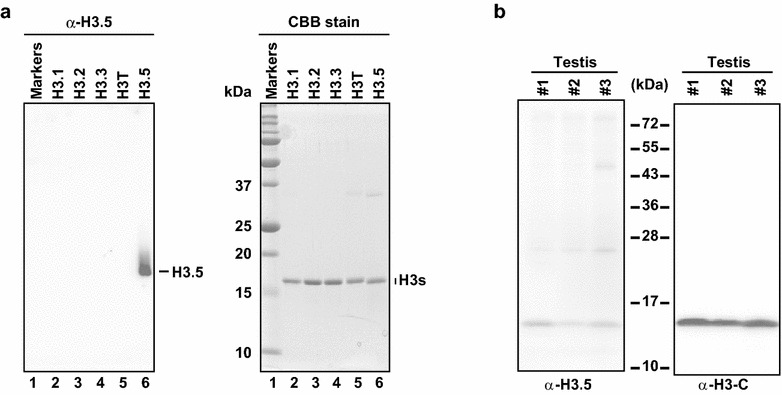
The H3.5-specific antibody. **a** Western blot analysis of H3.5. Purified H3.1, H3.2, H3.3, H3T, and H3.5 were separated by 18 % SDS-PAGE, and analyzed by Western blotting with the anti-H3.5 monoclonal antibody (*left panel*). The 18 % SDS-PAGE gel stained with Coomassie Brilliant Blue is presented in the right panel. **b** Western blot analyses of endogenous H3.5 (*left panel*) and all H3 s (*right panel*). Endogenous H3.5 and H3 proteins were detected by Western blotting with the anti-H3.5 monoclonal antibody (*left panel*) and the H3 C-terminus-specific antibody (*right panel*), respectively

**Fig. 7:**
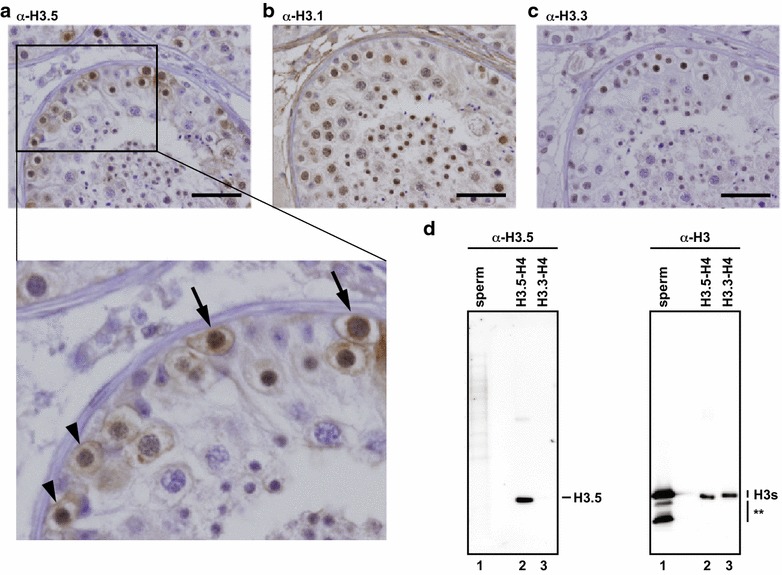
H3.5 exists in human testicular cells within seminiferous tubules. **a**–**c** Human testis sections immunohistochemically stained with the anti-H3.5 (**a**), anti-H3.1 (**b**), and anti-H3.3 (**c**) monoclonal antibodies. *Bars* indicate 50 μm.* Arrows* and* arrow heads* in the enlarged picture in* panel* (**a**) primary spermatocytes at leptotene and preleptotene stages, respectively. **d** Western blotting. Proteins from isolated sperm were separated by 15 % SDS-PAGE, transferred to a PVDF membrane, and probed with the anti-H3.5 monoclonal antibody (*left panel*) or the anti-H3 C-terminal peptide polyclonal antibody (*right panel*). Recombinant H3.5-H4 and H3.3-H4 complexes were used for controls. *Asterisks* represent the degraded H3

Since the endogenous H3.5 protein has not been detected, due to the lack of a specific antibody, we generated a specific monoclonal antibody directed against H3.5, using a peptide containing H3.5 Thr22-Arg41 (MAB Institute, Inc.). A Western blotting analysis showed that the H3.5 antibody specifically reacted to H3.5, but not to other variants (i.e., H3.1, H3.2, H3.3, and H3T) (Fig. 6a). In addition, we performed a Western blotting analysis with human testicular cell extracts from three individuals, and confirmed that the H3.5 antibody specifically detected endogenous H3.5 with low background signals (Fig. 6b). By using this antibody in immunohistochemical analyses, we detected positive signals in human testis sections, indicating that H3.5 is expressed at the protein level in cells within seminiferous tubules (Fig. 7a). Major histone H3 variants, such as H3.1 and H3.3, were also detected in testis sections (Fig. 7b, c). Interestingly, H3.5 was clearly present in spermatogonia and/or primary spermatocytes, in which the first meiotic cell division is not completed (Fig. 7a). However, the endogenous H3.5 protein was not detected in mature sperm by Western blotting using the H3.5-specific antibody, although H3 was clearly detected when the C-terminus-specific antibody was used (Fig. 7d), suggesting that H3 variants other than H3.5 are present in mature sperm. These results prompted us to test the genomic localization of endogenous H3.5 in human testicular cells.

### Transcription independent accumulation of H3.5 around transcription start sites in human testis

**Fig. 8:**
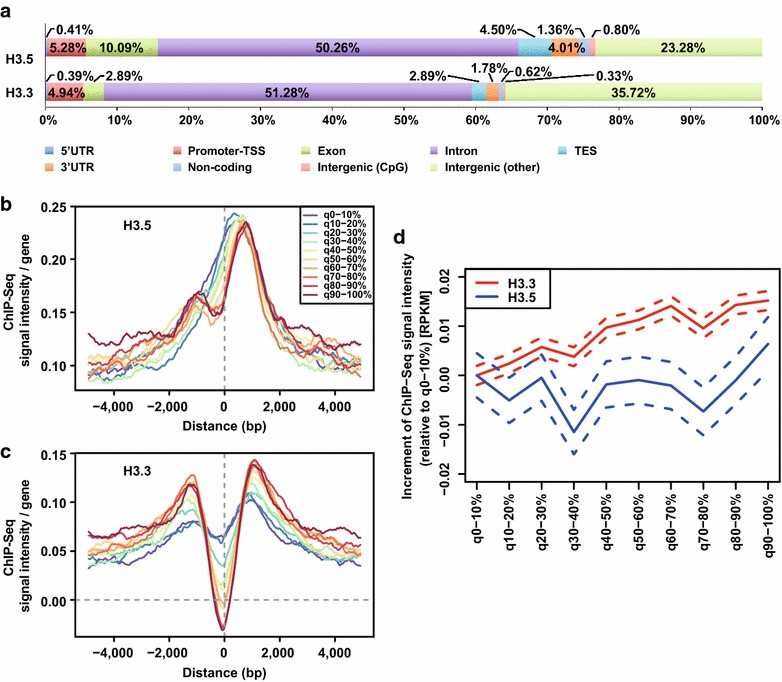
Endogenous H3.5 specifically accumulates around transcription start sites in human testicular cells. **a** Graphic representation of the distribution of endogenous H3.5 and H3.3 on the human genome. 5′-UTR: 5′-untranslated region. 3′-UTR: 3′-untranslated region. TSS: transcription start site. TES: transcription end site. **b** Aggregation plots of the endogenous H3.5 localization around TSSs. **c** Aggregation plots of the endogenous H3.3 localization around TSSs. Signals for genes with various expression levels are plotted against relative distance from TSS. **d** Gene expression-independent incorporation of endogenous H3.5 in human testis. The increment of each H3.3 and H3.5 ChIP-Seq signal intensity is shown with respect to the gene expression levels (per 10 %ile FPKM). The *red* and *blue lines* indicate the average increments of H3.3 and H3.5, respectively. The standard error (S.E.) of the signal intensities is indicated as a *dotted line*

To address the genomic incorporation pattern of endogenous H3.5, we performed chromatin immunoprecipitation coupled with deep sequencing (ChIP-Seq). We analyzed the distribution of H3.5 and H3.3 on the human genome. About 75 % of the H3.5 peaks were localized around genes (including 5′UTR, promoter-TSS, exon, intron, TES, and 3′UTR), as were the H3.3 peaks, although H3.5 was preferentially localized around exons, as compared to H3.3 (Fig. 8a). Additionally, H3.5 was enriched around transcription start sites (TSSs), especially downstream of TSSs, independently of its expression level (Fig. 8b). In contrast, the H3.3 distribution depended on its gene expression levels (Fig. 8c), as demonstrated previously [32]. Around TSSs, H3.5 was incorporated into both active and silent genes, while H3.3 was predominantly incorporated into active genes (Fig. 8d).

## Discussion

### Histone H3.5 in human seminiferous tubules

During spermatogenesis, the haploid genome becomes tightly packed into the sperm nucleus. This process requires robust chromatin reorganization, and eventually the histones are largely replaced by protamines [33–35]. Some human histone variants, including TSH2B and H3T, are highly expressed in testis, at least at the mRNA level, and may perform specific functions in the chromatin reorganization during spermatogenesis. H3.5 is a relatively newly identified variant, and its mRNA is also highly expressed in testis, as compared to other tissues [18]. However, the endogenous H3.5 protein has not been detected, due to the lack of its specific antibody. To overcome this problem, we successfully produced an H3.5-specific monoclonal antibody, which did not cross-react with other human H3 variants, H3.1, H3.2, H3.3, and H3T (Fig. 6). This H3.5 monoclonal antibody allowed us to study the endogenous H3.5 in human testis.

Our immunohistochemical analysis with human testis sections detected H3.5 at the protein level in seminiferous tubules, especially in the cells before or during the first meiotic cell division (Fig. 7). However, H3.5 may not be retained in mature sperm (Fig. 7). Therefore, H3.5 may play a role in preparing the proper chromatin landscape for events before or during the first meiotic cell division. Stage-specific production has been reported for the mouse testis-specific H2B variant, TH2B [36], which is an ortholog of human TSH2B [37]. However, the timing of TH2B expression is clearly different from that of H3.5. TH2B is barely detected in spermatogonia, but exists in spermatids. In contrast, H3.5 can be detected in spermatogonia, but not in mature sperm, although we cannot exclude the possibility that a trace amount of H3.5 is retained at limited genomic loci in human sperm. The stage-specific incorporation of different histone variants may play a role in the maturation of sperm chromatin.

### The nucleosome containing histone H3.5 is unstable

We found that the H3.5 nucleosome is quite unstable, as compared to the H3.3 nucleosome in vitro (Fig. 1). Consistently, the mobility of H3.5 is remarkably faster than that of H3.3 in living cells (Fig. 5). In humans, the expression level of another histone H3 variant, H3T, is also high in the testis, but low in somatic cells [6, 8, 9]. The nucleosome containing H3T is quite unstable in vitro and in living cells [26]. Nucleosome instability was also reported with a mouse testis-specific H2A variant, H2AL2 [38]. Therefore, instability may be a common characteristic of the testis-specific nucleosomes. The unstable nature of the H3.5 nucleosome may be suitable for further replacement with transition proteins and protamines. H3.5 incorporation may also regulate the transcription of the genes required during the early stages of spermatogenesis. However, H3.3 appears to be more relevant for regulating transcription during spermatogenesis, as its incorporation is correlated with the gene expression level. In contrast, H3.5 incorporation may function to transiently mark TSSs to assist in the replacement with H3.3, depending on gene expression. Histone acetylation may also play an important role in global and/or local histone exchange in the human testis, as shown in the mouse [39].

Our present and previous [26] studies demonstrated that the H3.5 Leu103 and H3T Val111 residues are predominantly responsible for the instability of the H3.5 and H3T nucleosomes, respectively. These H3.5 Leu103 and H3T Val111 residues correspond to Phe and Ala in H3.3 (and canonical H3.1), respectively. In the crystal structure of the H3.5 nucleosome, the H3.5 Leu103 residue forms fewer hydrophobic interactions with H4, as compared to the corresponding H3.3 Phe104 residue, and does not induce substantial structural distortion around the residue (Fig. 2). In contrast, the H3T Val111 residue induces local structural distortion around position 111 [26]. Therefore, the H3.5 Leu103 and H3T Val111 residues induce nucleosome instability by different mechanisms.

The H3.5 Leu103 and H3T Val111 residues are both located in the vicinity of the nucleosomal dyad. Intriguingly, genetic and biochemical studies have identified a mutation at Arg116 (to His) that destabilizes the nucleosome [40, 41]. This H3 mutation is known as a Sin mutation that alleviates the requirement for the Swi/Snf nucleosome-remodeling factor [40, 42]. The Sin phenotype has also been found in Saccharomyces cerevisiae with the H3 Ala111 to Gly mutation [43]. In addition, comprehensive alanine-scanning mutagenesis in *S. cerevisiae* suggested that the mutation of the 103rd or 104th residue of H3 may affect transcriptional regulation, probably through chromatin remodeling [44]. The crystal structure of the nucleosome containing the Sin mutations revealed that the H3 Arg116 mutation may allosterically destabilize the nucleosome, by reducing the number of histone-DNA and/or histone–histone interactions [45]. Therefore, the H3 C-terminal region, which is located near the nucleosomal dyad, is important for stable nucleosome formation, and amino acid substitutions within this region sensitively affect the nucleosome stability.

In addition to the testis, small amounts of H3.5 mRNA expression are also observed in ejaculate, leukocytes, and liver [18]. Proteins specifically produced in the testis have frequently been found as inappropriately overexpressed proteins in cancer cells. Furthermore, several missense mutations of the human *H3F3C* gene, encoding H3.5, have recently been found in tumors [46], including Val100 and Arg130. Like the L103F and Sin mutations, the mutations of these residues may also influence the nucleosome stability, by affecting the intra-nucleosomal interactions of amino acid residues near the nucleosomal dyad [40–45]. Together, these findings suggest that the inappropriate production of the H3.5 mutant may compromise proper chromosomal function in tumor cells. It is thus intriguing to study the correlation between H3.5 nucleosome stability and cancer predisposition.

## Conclusions

We found that the H3.5 nucleosome is less stable than the H3.3 nucleosome, and the H3.5-specific Leu103 residue is responsible for the instability of the H3.5 nucleosome, both in vitro and in living cells. We discovered that the H3.5 protein is actually present in seminiferous tubules in humans. Although the sample was limited to a single donor, our ChIP-seq analysis suggests that the endogenous H3.5 specifically accumulates at transcription start sites in human testicular cells. These findings provide new important insights into the role of H3.5 during spermatogenesis. Future analyses using more specimens from various donors, including those suffered from diseases, will be required for fully understanding the function of H3.5 in the chromatin reorganization.

## Methods

### Expression and purification of recombinant human histones

Human histones H2A, H2B, H3.1, H3.2, H3.3, H3T, H3.5, and H4 were produced in Escherichia coli cells [47], and were purified by the previously described method [26]. The human histone H3.5 vector for the *E. coli* expression system was constructed by site-directed mutagenesis, with the H3.3 expression vector as the template. The H3.3 and H3.5 mutants were also constructed by site-directed mutagenesis, and were prepared by the previously described method [26].

### Preparation of histone complexes

Freeze-dried H2A (2 mg), H2B (2 mg), H4 (1.6 mg), and either H3.5 (2.2 mg), H3.3 (2.2 mg), or H3.1 (2.2 mg) were mixed in unfolding buffer (20 mM Tris–HCl (pH 7.5), 7 M guanidine hydrochloride, and 20 mM 2-mercaptoethanol), and dialyzed against refolding buffer (10 mM Tris–HCl (pH 7.5), 2 M NaCl, 1 mM EDTA, and 5 mM 2-mercaptoethanol). The resulting histone octamers were purified by Superdex 200 gel filtration chromatography (GE Healthcare).

The H3.5-H4, H3T-H4, H3.3-H4, and H2A-H2B complexes were reconstituted as follows. Purified H3.5, H3T, or H3.3 was combined with H4 at a 1:1 molar ratio in unfolding buffer, and the mixture was dialyzed against refolding buffer, followed by stepwise salt-dialysis with refolding buffer containing 1, 0.5, and 0.1 M NaCl. Purified H2A and H2B were mixed at a 1:1 molar ratio in unfolding buffer, and the mixture was dialyzed against refolding buffer. The resulting H3.5-H4, H3T-H4, H3.3-H4, and H2A-H2B complexes were purified by Superdex 200 gel filtration chromatography.

### Preparation of nucleosomes

The nucleosomes were reconstituted using the 146 base-pair human α-satellite DNA [2], prepared by the method described previously [48]. For the H3.5, H3.3, and H3.1 nucleosomes, the purified histone octamers were mixed with the 146 base-pair DNA in a solution containing 2 M KCl. For the H3T nucleosome, the H3T-H4 and H2A-H2B complexes were mixed with the 146 base-pair DNA in a solution containing 2 M KCl [26]. The nucleosomes were reconstituted by the salt-dialysis method, heated at 55 °C for 2 h, and further purified from the free DNA and histones by non-denaturing polyacrylamide gel electrophoresis (Prep Cell, Bio-Rad).

### Salt resistance assay for nucleosome stability

The nucleosomes (240 ng/μl) were incubated in the presence of 0.4, 0.6, 0.7, and 0.8 M NaCl in 36 mM Tris–HCl (pH 7.5) buffer, containing 1.8 mM EDTA and 1.8 mM dithiothreitol, at 50 °C for 1 h. After this incubation, the NaCl concentrations of the samples were adjusted to 0.4 M, and the samples were analyzed by non-denaturing 6 % PAGE with ethidium bromide staining.

### Crystallization and structure determination

The crystallization and structural determination of the H3.5 nucleosome were performed by methods similar to those reported previously [25–27]. Crystals of the purified H3.5 nucleosomes were obtained by the hanging drop vapor diffusion method. The drop included the H3.5 nucleosome (1 μl) and a solution (1 μl) containing 20 mM potassium cacodylate (pH 6.0), 50 mM KCl, and 75–155 mM MnCl_2_. The reservoir solution contained 20 mM potassium cacodylate (pH 6.0), 35–40 mM KCl, and 50–80 mM MnCl_2_. Crystals typically appeared within 7–10 days, and grew to their full size over a period of 2–3 weeks. The H3.5 nucleosome crystals were soaked for 5–10 s at room temperature in a cryo-protectant solution, containing 20 mM potassium cacodylate (pH 6.0), 40 mM KCl, 70 mM MnCl_2_, 29 % 2-methyl-2,4-pentanediol, and 2 % trehalose, and were flash-cooled in a stream of N_2_ gas (100 K). The H3.5 nucleosome crystals contained one nucleosome per asymmetric unit. Diffraction data were obtained at a wavelength of 1.000 Å, using the synchrotron radiation source at the beamline BL41XU station of SPring-8, Harima, Japan.

Diffraction data were integrated and scaled with the HKL2000 program [49]. The data were processed using the CCP4 program suite [50]. The structure of the H3.5 nucleosome was solved by the molecular replacement method, using PHASER [51] and the H3.3 nucleosome structure [PDB:3AV2] as the search model [27]. The structure of the H3.5 nucleosome was refined using PHENIX [52], and the model was built using COOT [53]. Following the rigid body refinement, iterative rounds of xyz-coordinate, real-space, individual B-factor, and occupancy refinements were performed, with optimizing the X-ray/stereochemistry and the X-ray/B-factor weights. Secondary structure restraints and non-crystallographic symmetry restraints were applied for the refinements. The Ramachandran plot of the final H3.5 nucleosome structure showed 99.2 % of the residues in the favored region, 0.8 % of the residues in the allowed region, and no residues in the outlier region, as validated with the MolProbity program [54]. A summary of the data collection and refinement statistics is provided in Table 1. All structure figures were created using the PyMOL program [55].

### Thermal stability assay

The tetrasomes were reconstituted by the salt dialysis method, and were purified by non-denaturing polyacrylamide gel electrophoresis (Prep Cell, Bio-Rad), as previously described [28]. The reconstituted tetrasomes, containing the H3.5-H4 or H3.3-H4 tetramer and the 146 base-pair DNA, were subjected to the thermal stability assay, in 19 µl of 20 mM Tris–HCl (pH 7.5), 1 mM DTT, and 1 mM EDTA. The fluorescence signals were detected by a StepOnePlus™ Real-Time PCR unit (Applied Biosystems) with a temperature gradient from 26 to 95 °C, in steps of 1 °C/min. The fluorescence intensity was normalized, as follows: F(T)_normalized_ = [F(T) − F(26)]/[F(95) − F(26)], and plotted against the temperature. F(T) indicates the fluorescence intensity at a particular temperature.

### FRAP analysis

DNA fragments encoding H3.5, H3.3, and their mutants were cloned into the pEGFP-C3 vector (Clontech). HeLa cells were transfected with the expression vectors using Lipofectamine 2000 (Life Technologies), and cultured in 1 mg/ml G418 (Nacalai Tesque) to select those stably expressing GFP-tagged H3 proteins. Cells were grown in Dulbecco’s modified Eagle’s medium, supplemented with 10 U/ml penicillin, 50 μg/ml streptomycin, and 10 % fetal calf serum, on a glass-bottom dish (Mat-tek). FRAP was performed using a confocal microscope (FV-1000; Olympus), featuring a heated stage supplemented with 5 % CO2 [29]. A confocal image of a field containing about 10 nuclei was collected with a 60×  UPlanSApo NA = 1.35 lens (800 × 800 pixels, zoom 2, scan speed 2 μs/pixel, pinhole 800 μm, Kalman filtration for four scans, LP505 emission filter, and 0.2 % transmission of 488-nm Ar laser). Afterward, one-half of each nucleus was photobleached using 90 % transmission of the 488 nm laser (two iterations), and images were collected using the original setting at 1 min intervals for 120 min. Fluorescence intensities of the bleached, unbleached, and background areas were measured using Image J 1.46r. After background subtraction, the relative intensity of the bleached area to the unbleached area was determined and normalized to the initial value before bleaching.

### The H3.5 monoclonal antibody

The H3.5-specific mouse monoclonal antibody was produced (MAB Institute Inc.) using an H3.5-specific synthetic peptide (TKAARKSTPSTCGVKPHRYRC) coupled to keyhole limpet hemocyanin. After hybridoma generation, clones were screened by ELISA, using plates coated with the H3.5-specific or H3.3-specific (TKAARKSAPSTGGVKKPHRYRC) peptide conjugated with bovine serum albumin. ELISA-positive clones were further screened by immunofluorescence using HeLa cells expressing GFP-H3.5 and GFP-H3.3, and by Western blotting using recombinant histone H3 variants (i.e., H3.1, H3.2, H3.3, H3T, and H3.5).

### Immunohistochemistry

Human testicular samples were obtained, fixed in Bouin’s solution for 2 h and embedded in paraffin. After deparaffinization, 5-μm sections were incubated with hydrogen peroxide to inhibit endogenous peroxidases. After non-specific binding was blocked by rabbit serum, the slides were incubated with the culture supernatant of the hybridoma producing the anti-H3.5 monoclonal antibody (1:100 dilution) for 24 h at room temperature, and endogenous H3.5 was visualized using the avidin–biotin complex method. The sections were then counterstained with haematoxylin. In the negative control slides, the primary antibody was omitted. Endogenous H3.1 and H3.3 were detected by the same method as that for the H3.5 detection, using the H3.1-specific monoclonal antibody [56] or the H3.3-specific monoclonal antibody [57].

### Detection of histone H3 in mature sperm cells

Ejaculated sperm cells were obtained with informed consent from 12 anonymized Japanese males. Mixed sperm cells were washed 3 times with PBS, and were suspended in lysis buffer (20 mM Tris–HCl (pH 8.0), 10 % glycerol, 0.2 mM EDTA, 0.1 % Tween 20, 150 mM KCl, 0.2 mM PMSF, and 0.8 mM 2-mercaptoethanol). The collected samples were sonicated six times for 30 s in lysis buffer. The proteins in the cell lysate were fractionated by 15 % SDS-PAGE, transferred to a PVDF membrane, and detected with an anti-H3 rabbit polyclonal antibody (#9715, Cell Signaling Technology, Inc.) or the culture supernatant of the hybridoma producing the anti-H3.5 monoclonal antibody (1:100 dilution).

### Chromatin immunoprecipitation

Human testis homogenates were fixed with 1 % formaldehyde in PBS(+) buffer. The fixed human testis homogenates were precipitated then suspended in RIPA buffer (50 mM Tris–HCl (pH 8.0), 150 mM NaCl, 2 mM EDTA, 1 % NP-40, 0.5 % sodium deoxycholate, 0.1 % SDS, and protease inhibitor cocktail; Nacalai Tesque Inc.), instead of ChIP buffer. The sample was sonicated twenty times for 5 s. The sheared samples were then centrifuged at 15,000×*g* for 10 min. The supernatant, containing the DNA, was incubated with magnetic beads conjugated with the anti-H3.3 rat monoclonal antibody [57] or the culture supernatant of the hybridoma producing the anti-H3.5 mouse monoclonal antibody, at 4 °C overnight with rotation. The immune complexes were pulled down, washed with RIPA buffer and TE buffer (both twice), and then eluted from the beads using 1 % SDS and 0.02 % Proteinase K (Nacalai Tesque Inc.) in TE. The cross-links were reversed by an incubation for 4 h at 65 °C, followed by an incubation for 1 h at 50 °C. The DNA samples were then purified with a Qiaquick PCR purification kit (Qiagen, Valencia, CA, USA). The ChIP library was prepared with the Illumina protocol, and the samples were sequenced on an Illumina HiSeq-1500 system.

### ChIP-Seq data analysis

Sequenced reads of H3.3 and H3.5 ChIP-Seq were mapped onto the human genome (hg19) with Bowtie (version 0.12.8), with the parameters “–v 2 –m 1”. The uniquely mapped and PCR duplicates-removed reads, obtained using SAMTools [58], were utilized for further analysis. The number of reads for input, H3.5, and H3.3 were 26,524,770, 14,818,772, and 24,356,280, respectively. The estimation of normalized ChIP-Seq signal intensities was calculated as follows. First, we counted the mapped reads throughout 1000 bp intervals (bins) on the genome, and then the counts were normalized as RPKM (Reads Per Kilobases per Million reads) [59]. Finally, the ChIP-Seq signal intensities were calculated as the RPKM differences between the ChIP and input DNA-control data (i.e., ChIP–control) on each bin. For the peak distribution analysis (percentages of peak localizations on each genomic category), the HOMER software [60] was utilized. Peaks were called using the MACS2 software (version 2.1.0) [61] with the following parameters: *q* value <0.1 for H3.5, *p* value <0.01 for H3.5.

### mRNA-Seq and analysis

Total RNA was extracted from human testis homogenates. cDNA synthesis was performed with Primescript Reverse Transcriptase and a dT primer (Takara Bio Inc.). The preparation of the mRNA-Seq library and the sequencing were performed according to the Illumina protocol. Sequenced reads were mapped onto the human genome (hg19) with Tophat (version 2.0.8). The gene expression levels (FPKM; Fragments Per Kilobase of exon per Million mapped sequence reads) were estimated with Cufflinks (version 2.0.1), using the mapped reads. The default parameters of the software were employed. We defined ten expression groups, labeled q0–10 %, q10–20 %,…, q90–100 %, with respect to the FPKMs of genes, which define the 10 percentile intervals of all FPKMs; i.e., the genes were ordered by the FPKMs and then separated into ten groups with equal numbers of members. The genes with FPKM = 0 were excluded.

### Data access

The atomic coordinates of the H3.5 nucleosome have been deposited in the RCSB Protein Data Bank, with the RCSB ID code [PDB:4Z5T]. Deep-sequencing data have been deposited in the DDBJ sequence read archive, with the accession number [DDBJ:DRA002604].

## Abbreviations

TSS: transcription start site
GFP: green fluorescent protein
ChIP: chromatin immunoprecipitation
FRAP: fluorescence recovery after photobleaching
RPKM: reads per kilobases per million reads
FPKM: fragments per kilobase of exon per million mapped sequence reads
NCP: nucleosome core particle
CBB: Coomassie Brilliant Blue
